# Enhancing photoelectrochemical CO_2_ reduction with silicon photonic crystals

**DOI:** 10.3389/fchem.2023.1326349

**Published:** 2023-12-19

**Authors:** Chu Zhou, Gaotian Zhang, Peiyuan Guo, Chenxi Ye, Zhenjun Chen, Ziyi Ma, Menglong Zhang, Jingbo Li

**Affiliations:** ^1^ School of Engineering, University of Warwick, Coventry, United Kingdom; ^2^ Zhejiang Xinke Semiconductor Co., Ltd., Hangzhou, Zhejiang, China; ^3^ School of Semiconductor Science and Technology, South China Normal University, Foshan, Guangdong, China; ^4^ College of Optical Science and Engineering, Zhejiang University, Hangzhou, Zhejiang, China

**Keywords:** Si photonic crystal, photocatalyst, photoelectrochemistry, photocathode, CO_2_ reduction

## Abstract

The effectiveness of silicon (Si) and silicon-based materials in catalyzing photoelectrochemistry (PEC) CO_2_ reduction is limited by poor visible light absorption. In this study, we prepared two-dimensional (2D) silicon-based photonic crystals (SiPCs) with circular dielectric pillars arranged in a square array to amplify the absorption of light within the wavelength of approximately 450 nm. By investigating five sets of n + p SiPCs with varying dielectric pillar sizes and periodicity while maintaining consistent filling ratios, our findings showed improved photocurrent densities and a notable shift in product selectivity towards CH_4_ (around 25% Faradaic Efficiency). Additionally, we integrated platinum nanoparticles, which further enhanced the photocurrent without impacting the enhanced light absorption effect of SiPCs. These results not only validate the crucial role of SiPCs in enhancing light absorption and improving PEC performance but also suggest a promising approach towards efficient and selective PEC CO_2_ reduction.

## 1 Introduction

The widespread concern caused by global climate change has led to the rapid development of carbon-neutral strategies (Muradov and TNJIjohe, 2008; Zhao and You, 2020; Wang et al., 2021a). In these strategies, the efficient conversion and utilization of CO_2_ play a crucial role in the fields of carbon cycle and sustainable energy storage (He et al., 2022). Photoelectrochemistry (PEC) offers a potential solution by converting CO_2_ into economically valuable chemicals (Zhang et al., 2014a; Cheng et al., 2014; Kuk et al., 2017; Chu et al., 2018). This approach not only mitigates the environmental impact of fossil fuel use but also provides a strategy for solar energy storage.

Solar-driven PEC systems for CO_2_ reduction have been extensively studied (Cheng et al., 2020; Lu et al., 2020), in which the semiconductor material typically serves as the light-harvesting component. It converts absorbed photons into charge carriers that drive CO_2_ reduction reaction. In order to optimize this process, an efficient light absorber is required for effectively utilization of visible light part of the solar spectrum (Wu et al., 2019; Wang et al., 2021b). This typically could be achieved through a variety of strategies, such as doping (Huang et al., 2022), constructing heterostructures (Liu et al., 2018), and applying surface plasmon enhancement effects (Zhang et al., 2019).

In recent years, photonic crystals (PCs) have attracted widespread attention for the directional control of the propagation of light (Baba, 2008; Ishizaki and Noda, 2009; González-Urbina et al., 2012; Wu et al., 2018). The concept was first introduced in 1987 by Yablonovitch (Eli, 1987) and John (John SJPrl, 1987). Local control and manipulation of light waves could be achieved through the periodical arrangement of two dielectric materials, thereby significantly improving the intensity and lifetime of the internal light field. This characteristic provides an alternative strategy for optimizing the light absorption of PEC systems (Zhang et al., 2013; Zhang et al., 2014b; Fang et al., 2016; Li et al., 2019a). Efficient absorption of light of specific wavelengths could be achieved by accurately designing the band gap and mode of the photonic crystal, and on this basis, the efficiency of the photoelectrochemical process can be improved.

Silicon (Si) and silicon-based materials have gained popularity in the field of photoelectrochemistry due to their exceptional electronic properties (Shi et al., 2011; Duan et al., 2014; Tang et al., 2018; Hsiao et al., 2019; Putwa et al., 2023). However, silicon has inherent limitations in its response to visible light (Wang et al., 2012), such as bandgap restrictions and rapid recombination of photogenerated carriers. To overcome these limitations, researchers are employing nanoengineering and surface modification strategies, including nanostructure design (Priolo et al., 2014), surface carrier transport layers (Richter et al., 2021), and catalyst loading (Kempler et al., 2018). For instance, silicon nanowires (Roh et al., 2022), as a one-dimensional derivative of silicon, offer advantageous conditions for enhanced light absorption and improved carrier separation and migration efficiency. Nonetheless, there are challenges in further extending the light response range of silicon-based materials and efficiently utilizing solar energy. In this context, silicon photonic crystals, with their ability to precisely manipulate light propagation characteristics, present an interesting and potential solution (Ali et al., 2018; O’Brien et al., 2018).

In this study, a systematic approach was utilized to probe the efficacy of two-dimensional (2D) silicon-based photonic crystals with circular dielectric pillars arranged in a square array in advancing PEC CO_2_ reduction. We engineered five sets of n^+^p-SiPCs by typical lithography-etching patterning methods, each maintaining a consistent filling ratio while featuring different dielectric pillar sizes, with the prime objective of identifying the configuration that yields appreciable photocurrent density. Notably, we observed enhanced absorption at approximately 450 nm, a wavelength band that is substantially represented in the solar spectrum. This pivotal discovery is attributed to our ability to position the photonic band gap at the target wavelength by finely tuning the periodic structure and dielectric pillar sizes of the silicon photonic crystals. This allows for efficient absorption of light at a specific wavelength. Integrating electrochemically deposited platinum nanoparticles, we extended our investigation towards the selectivity of reduction products. Our key findings demonstrate a notable enhancement in light absorption and a captivating shift in product selectivity towards CH_4_ (∼25% FE). These results highlight a promising avenue for utilizing silicon photonic crystals in achieving efficient and selective photoelectrochemical CO_2_ reduction.

## 2 Materials and methods

### 2.1 Fabrication of n-Si epitaxy

4″ p-Si wafers with a resistivity of 1–30 Ω cm, 550 μm thickness and <100>-orientation were used as the substrate for the fabrication process. Silicon wafer was first submerged in piranha solution for 5 min to thoroughly remove any metallic and organic contaminants from the surface. Following this, the wafer was placed in buffered oxide, etch solution for 2 min to remove the silicon surface’s oxide layer. Subsequently, utilizing the Metal-Organic Chemical Vapor Deposition (MOCVD) apparatus (VEECO K465i), and choosing tert-butyl phosphine as the dopant source, a 5 μm single-crystal n-Si epitaxial layer was deposited on the silicon wafer.

### 2.2 Fabrication of n^+^p-silicon photonic crystals

A 2 µm thick positive photoresist was spin-coated onto the n-type side of the above silicon wafers. Subsequently, a Nikon i-Line Stepper lithography system was employed to create a square array of circular patterns using a mask. The mask was configured to have five groups of patterns with different periods of 5, 6.25, 7.5, 8.75, and 10 μm, resulting in photoresist patterns with periods of 1, 1.25, 1.5, 1.75, and 2 µm. The exposure time under UV light was adjusted to achieve targeted radii for the final resist circular patterns. The developed process involved using a positive developer and baking the photoresist pattern at 110°C for 90 s. An Inductively Coupled Plasma (ICP) etching system with O_2_ and SF_6_ as the etching gases was then used to, etch a dielectric pillar array structure. Finally, any remaining photoresist was removed using a photoresist stripper. The five groups of silicon photonic crystals (SiPCs) obtained were cleaned with deionized water and dried.

### 2.3 Electrochemical deposition of Pt nanoparticles

In preparation for the electrochemical deposition of platinum nanoparticles, the SiPCs underwent a thorough cleaning process using acetone, isopropyl alcohol, buffered oxide, etch solution, and deionized water. The platinum nanoparticles were then deposited electrochemically from a solution containing 5 mM H_2_PtCl_6_ and 0.5 M H_2_SO_4_. This deposition process took place in a conventional electrochemical cell equipped with a saturated calomel electrode (SCE) as a reference and a platinum plate serving as a counter electrode. The potentiostatic deposition of Pt occurred at −0.34 V vs. SCE (Domínguez-Domínguez et al., 2008). Throughout the entire process, all conditions were monitored using a CHI 760E electrochemical workstation (CH Instruments, Inc).

### 2.4 Photoelectrochemical CO_2_ reduction

A three-electrode system was applied for PEC CO_2_ reduction, consisting of a Pt counter electrode and a reference electrode of Ag/AgCl. SiPCs as well as n^+^p Si wafer, serving as the work electrode, were mounted on a platinum plate, with an exposed area of 0.32 cm^2^ facing the illumination window. The cell was filled with CO_2_-saturated 0.1 M KHCO_3_ electrolyte at a pH of 6.8, and CO_2_ was continuously supplied during the reduction process. All the PEC measurements were carried out on the CHI 760E electrochemical station (CH Instruments, Inc.) under ambient conditions. Irradiation was provided by a 300 W Xe lamp equipped with an AM 1.5G filter (Perfectlight, China). To convert the electrode potentials to values relative to the reversible hydrogen electrode (RHE), the Nernst Equation was employed with formula E (vs. RHE) = E (vs. Ag/AgCl) + 0.23 + (0.0591 * pH).

### 2.5 Characterization

The morphology of the SiPCs was characterized using a scanning electron microscope (SEM, Phenom Pharos G2 Desktop FEG-SEM). The steady-state surface photovoltage (SPV) spectra of the SiPCs were obtained through an SPV measurement system (PL-SPS 1000, Pefectlight) including a monochromatic light source. UV-vis spectra of the SiPCs were recorded on the UV-vis-nir spectrophotometer (UV-3600i plus, Shimadzu) in diffuse reflectance mode in the evaluation of light absorbance. The Pt nanoparticles were examined using transmission electron microscopy (TEM, JEM-2100F, JEOL, Ltd.) with EDX. Prior to TEM analysis, electrodeposited Pt nanoparticles were scraped off along with SiPC pillars from the Si substrate onto a copper grid covered with a carbon film. Gas products were collected by foil sample bags and manually injected into a gas chromatograph (GC, 7890B GC System, Agilent Technologies, Inc.) with a thermal conductivity detector and a flame ionization detector. The Faradaic efficiency (FE) was determined by dividing the total charge needed for each product by the total charge passed during the test. X-ray photoelectron spectroscopy (XPS, ThermoFisher Nexsa) was performed on the PtNPs@SiPCs photocathode before and after the PEC process. For XPS analysis, an Al Kα micro-focused X-ray source with a pass energy of 60 eV was used. The X-ray diffraction (XRD) spectra were collected by Cu Kα radiation on the Bruker D8 Advance diffractometer.

## 3 Results and discussions

**FIGURE 1:**
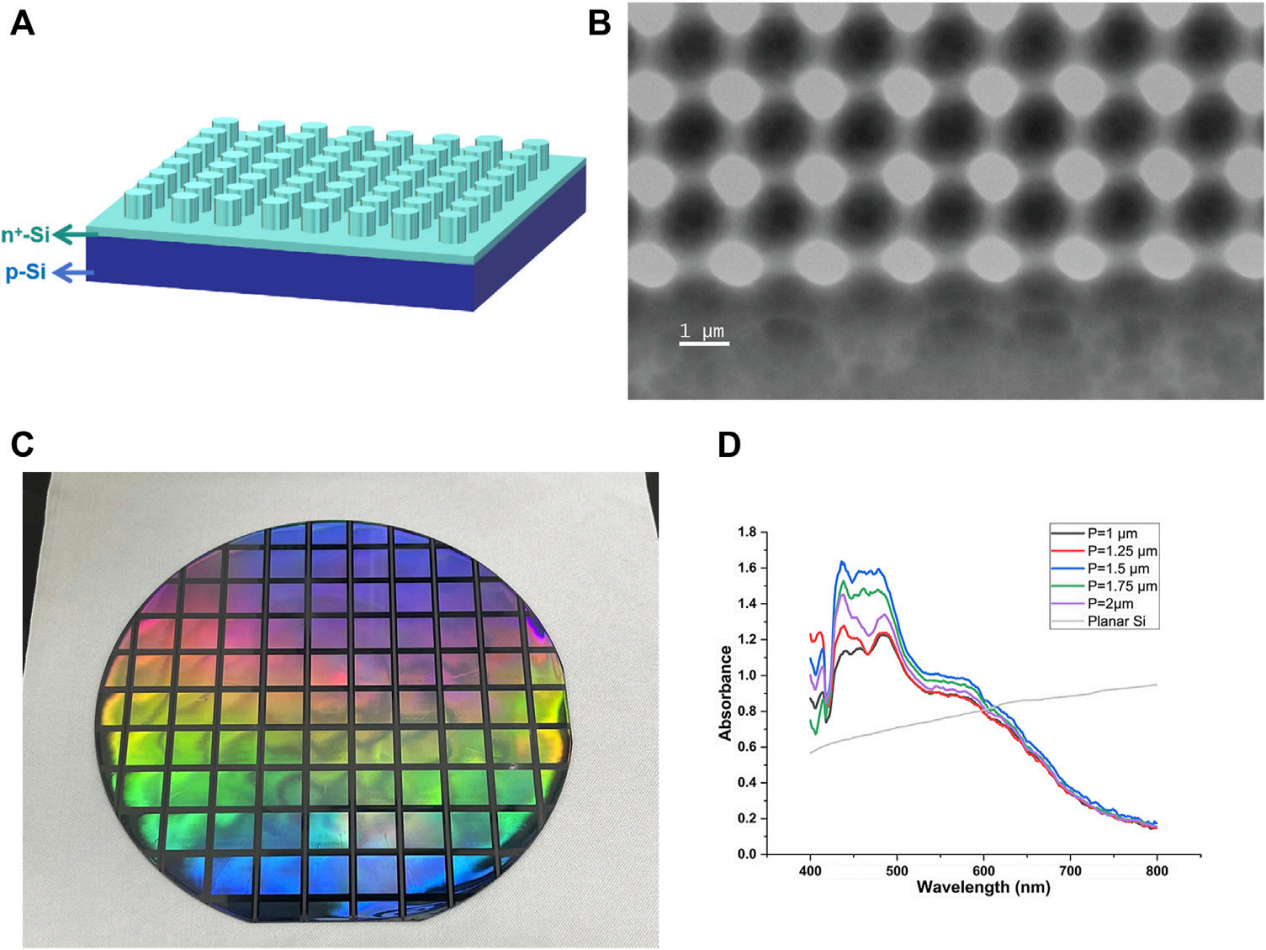
**(A)** Schematic of SiPCs. **(B)** SEM image of the SiPC. **(C)** Digital photo. **(D)** Absorption spectra.

Five groups of SiPCs were prepared by typical lithography-etching patterning methods. As described in the Method section, two-dimensional (2D) photonic crystals with circular dielectric pillars arranged in a square array were obtained (Figure 1A). While keeping the ratio of the pattern radius to the period (r/P, ∼0.3) and the height of the dielectric column unchanged, their periods (P) varied from 1 to 2 μm. The SEM images (Figure 1B) are consistent with the designed patterns. The structural colour (Figure 1C) displayed under illumination and the peak part of the absorption spectrum (Figure 1D) showed its modulation effect on a certain wavelength band (∼450 nm), further confirming the formation of photonic crystal structure.

**FIGURE 2:**
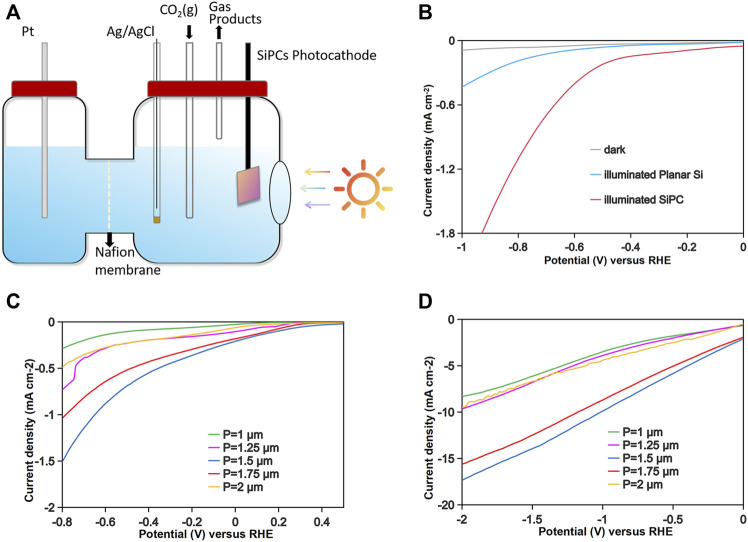
**(A)** Schematic of the PEC cell. **(B)** The J-V curves of the Si wafer and the SiPC with and without illumination. **(C)** The J-V curves of the SiPCs with different periodicity. **(D)** The J-V curves of the SiPCs with different periodicity after electrodeposition of Pt nanoparticles.

The PEC performances of these SiPCs were tested in the configuration shown in Figure 2A (see detailed conditions in Method section. Firstly, the SiPC with *p* = 1.5 μm was selected for comparison with a planar n^+^p-Si wafer. Figure 2B shows the LSV curves. In the absence of illumination, the current density is negligible for both the planar wafer and the SiPC. However, the introduction of the photonic crystal significantly enhances the current density under illumination. It should be pointed out that the n-Si epitaxy is much thicker than the height of the photonic crystal layer, resulting in shortened distance of carrier migration to the surface due to the removal of the silicon substrate for SiPC preparation is negligible. Consequently, the p-n junction plays an equal role in facilitating carrier separation, regardless of whether it is on SiPCs or the planar silicon wafer. Therefore, the observed increase in photocurrent densities can be attributed to the enhanced light absorption derived from the photonic crystal structure.

Then, we conducted tests on the five groups of SiPCs with different periodicities. The LSV curves (Figure 2C) reveal that the performance ranking among the groups is in good agreement with their absorption spectra. Among them, SiPC with a periodicity of 1.5 μm exhibits the highest photocurrent density. This suggests that the gradual increase in absorption capacity of SiPCs within the target wavelength band (∼450 nm) significantly contributes to the enhancement of photocurrent while there was minimal variation in absorption across other wavelengths (such as 600–800 nm).

**FIGURE 3:**
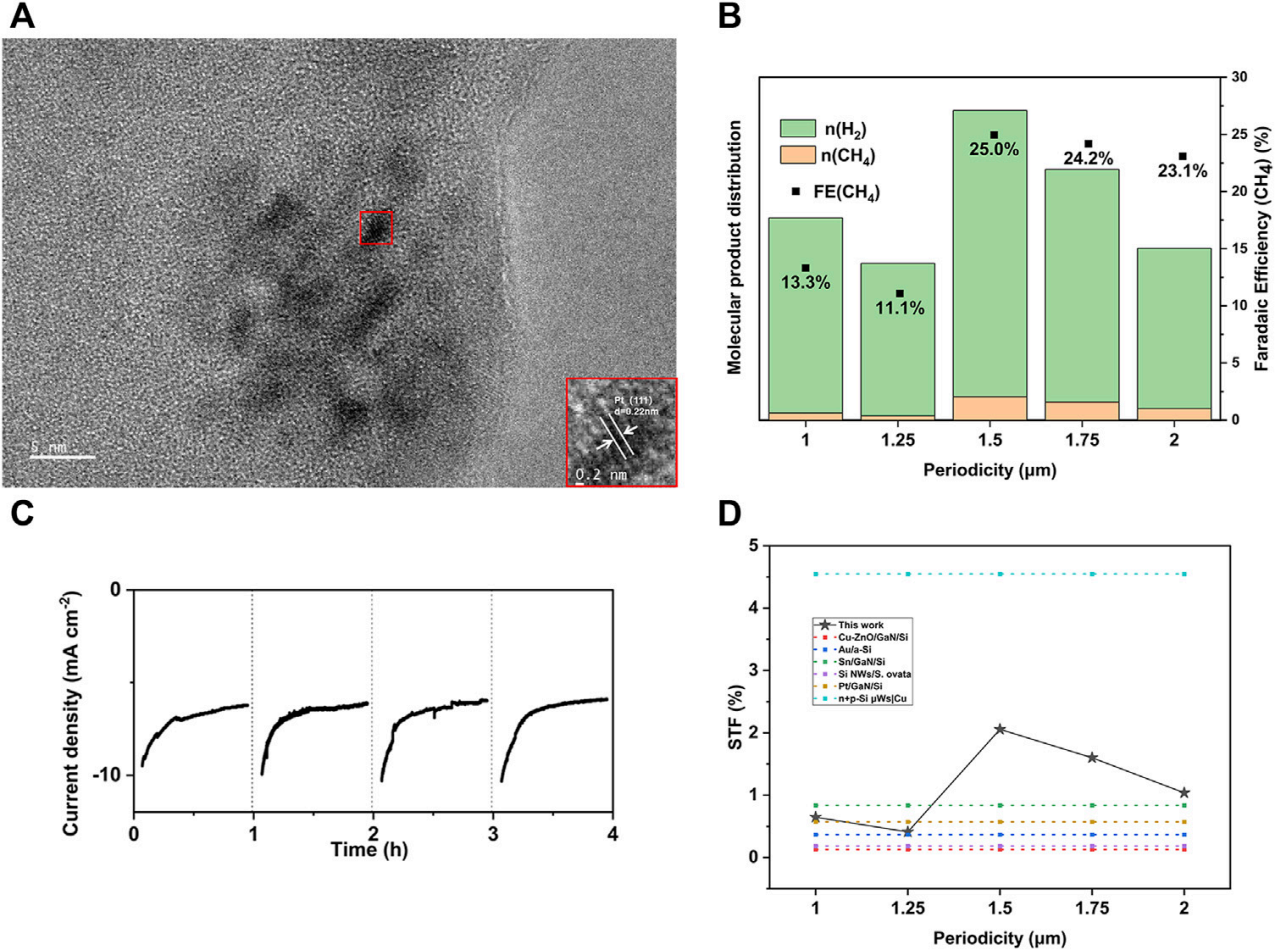
**(A)** TEM image illustrates Pt nanoparticles. **(B)** Comparison of the molecular product distribution and FE. **(C)** Chronoamperometry data of the Pt@SiPC (*p* = 1.5 μm) from 4 consecutive runs. **(D)** Performance comparison by STF of different Si-based photocathodes covered in other works and in this work. The periodicity is only relevant to SiPCs in this work and the STFs of other works were estimated from J-V curves and FEs accordingly.

Photocurrent density alone is not sufficient for evaluating photoelectrochemical efficiency, as there are competing reactions, particularly the hydrogen evolution reaction (HER) (Ross et al., 2019). Typically, a Si-based photocathode is combined with metal nanoparticles to drive the reaction towards high-value products. In this study, we conducted electrodeposition to assemble Pt nanoparticles (PtNPs) on SiPCs. To ensure consistency, we adjusted the electrodeposition time until the PEC performance was optimized, while excluding the influence of the number and distribution of Pt nanoparticles. Transmission electron microscopy (TEM) was utilized to characterize the PtNPs (Figure 3A). The LSV curves, as shown in Figure 2D, demonstrate that the introduction of PtNPs significantly enhances the photocurrent without altering the performance ranking among the SiPCs groups. This finding underscores the dominant role of photonic crystals in determining PEC performance, as the deposition of metal nanoparticles or any other catalysts on the SiPCs surface does not have a negative impact on the effect of photonic crystals.

The molecular distribution and Faradaic Efficiencies (FEs) of the gas products were obtained after the Pt@SiPCs worked for 1 h under a constant applied potential (−1.15 V versus RHE), as shown in Figure 3B. The PEC reduction of CO_2_ tended to produce a higher proportion of methane when it was catalyzed by the SiPC with greater photocurrent density. Notably, Pt@SiPCs with a period of 1.5 μm achieved the highest FE of 25%. This could possibly be attributed to the larger number of photogenerated electrons facilitating the conversion of CO_2_ to CH_4_, which is a reduction reaction requiring more electrons compared to HER (Zeng et al., 2020; Wang et al., 2021c). Besides, a more negative photogenerated potential may enhance the formation of reactive intermediates such as CO, leading to higher selectivity for CH_4_ (Meng et al., 2021).

Additionally, four repeated tests were conducted to evaluate the sustainability of samples. Electrolyte and CO_2_ were replenished between each run. The chronoamperometry data of the Pt@SiPC (*p* = 1.5 μm) from the four consecutive runs can be seen in Figure 3C. The achieved photocathode reliability was considered acceptable, given the overall similar performance in the four runs. The decrease in current density at the beginning of each run could possibly be attributed to the thermal effect caused by illumination. Higher operating temperatures decreased the solubility of CO_2_ in the solvent (Zhang et al., 2018). Upon reaching thermal equilibrium, the PEC system exhibited a stable photocurrent. By replenishing the CO_2_-saturated electrolyte between each run, the initial current density could be restored at the start of the subsequent test.The solar-to-fuel (STF) efficiency in terms of CH_4_ was considered in the evaluation of the Pt@SiPCs and was given by Eq. 1:

$$\eta_{STF}=\frac{P_{out}}{P_{in}}=\frac{J_{op}\frac{FE}{n_{p}F}LHV_{p}}{P_{Solar}}$$ (1)

Where *P*_*out*_ is the energy stored in the target product, *P*_*in*_ is the inlet power, *J*_*op*_ is the operation current density, *FE* is the Faradaic efficiency of the target product, *n*_*p*_ is the number of electrons transferred, *F* is the Faraday constant, *LHV*_*p*_ is the lower heating value per mole of the target product, and *P*_*Solar*_ is the power of the incident light per unit of area (Singh et al., 2015). As shown in Figure 3D, the optimized Pt@SiPCs achieved an impressive STF (∼2.1%) in comparison to Si-based photocathodes covered in existing research (Liu et al., 2015; Chu et al., 2016; Li et al., 2019b; Zhou et al., 2019; Kempler et al., 2020). However, there is still a performance gap with state-of-the-art Si photocathodes, as evidenced by the limited photocurrent density and the absence of C_2+_ products. This could be attributed to challenges in carrier separation and migration due to the planar p-n structure in our design. The selectivity of C_2+_ products is influenced by metal catalysts, which may also contribute to the performance gap. Additionally, the SiPC structure in this case has limitations in terms of specific surface area. It is important to note that optimizing these aspects does not conflict with the structure of the photonic crystal. By employing photonic crystals architecture in Si-based PEC systems, it is expected that the PEC CO_2_ reduction performance under Si-based photocathodes will experience significant improvement.

## 4 Conclusion

In this study, the two-dimensional n^+^p Si photonic crystals (SiPCs) were fabricated using a typical photolithography-etching process. The SiPCs consisted of a periodic circular dielectric pillar structure arranged in a square array, which exhibited a remarkable enhancement in absorption within the wavelength range of approximately 450 nm. Compared to planar n^+^p Si wafers, SiPCs demonstrated higher photocurrent density and catalytic activity. By adjusting the periodicity of the pattern arrangement, the modulation effect on the specific wavelength could be improved while maintaining the filling factor, resulting in increased photocurrent density. Further investigations revealed that the introduction of Pt nanoparticles facilitated CO_2_ reduction towards CH_4_ production in SiPCs, with selectivity for CH_4_ of up to 25%. Therefore, due to its ability to enhance light utilization and its unique plasticity, SiPCs hold significant potential in the field of photoelectrochemical CO_2_ reduction and possibly other photochemical reactions.

## Data Availability

The raw data supporting the conclusion of this article will be made available by the authors, without undue reservation.
