# Supplementary material for: Enhancing photoelectrochemical CO2 reduction with silicon photonic crystals
Source: Front Chem. 2023 Dec 19;11:1326349. doi: 10.3389/fchem.2023.1326349 (PMC10758474; doi:10.3389/fchem.2023.1326349)
Supplement: Supplementary file 1 [file DataSheet1.docx]

Supplementary information


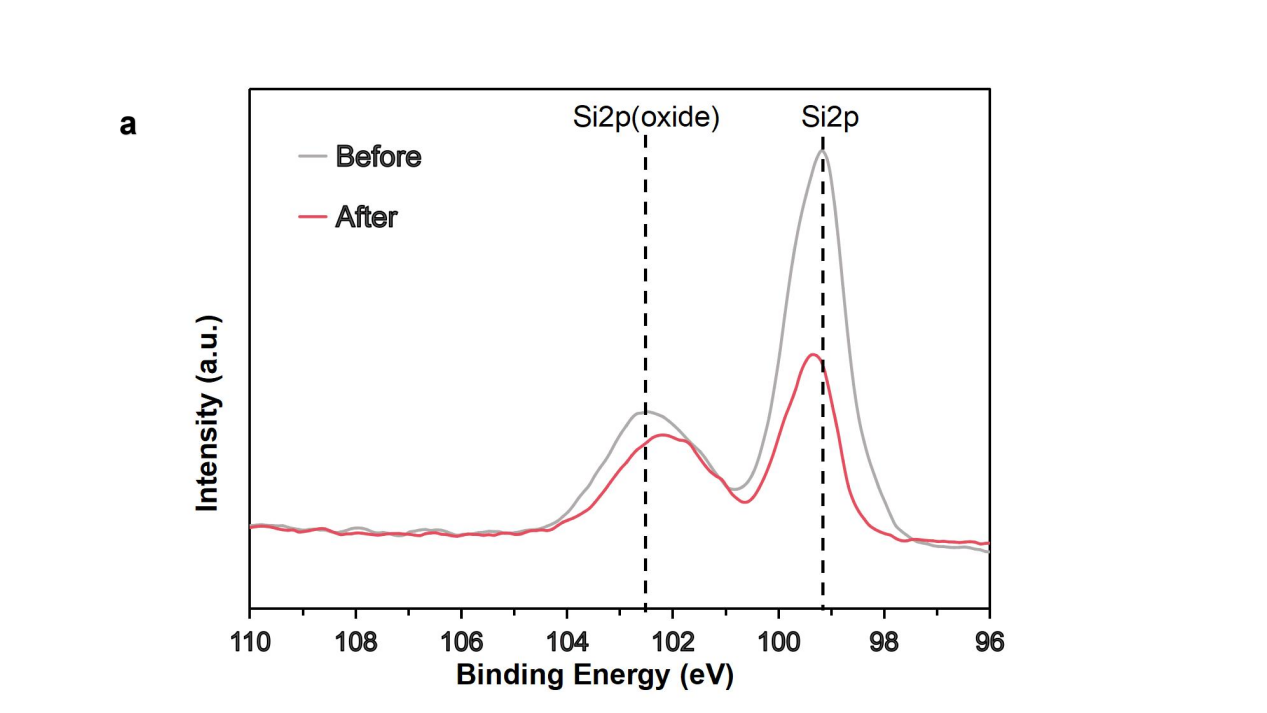

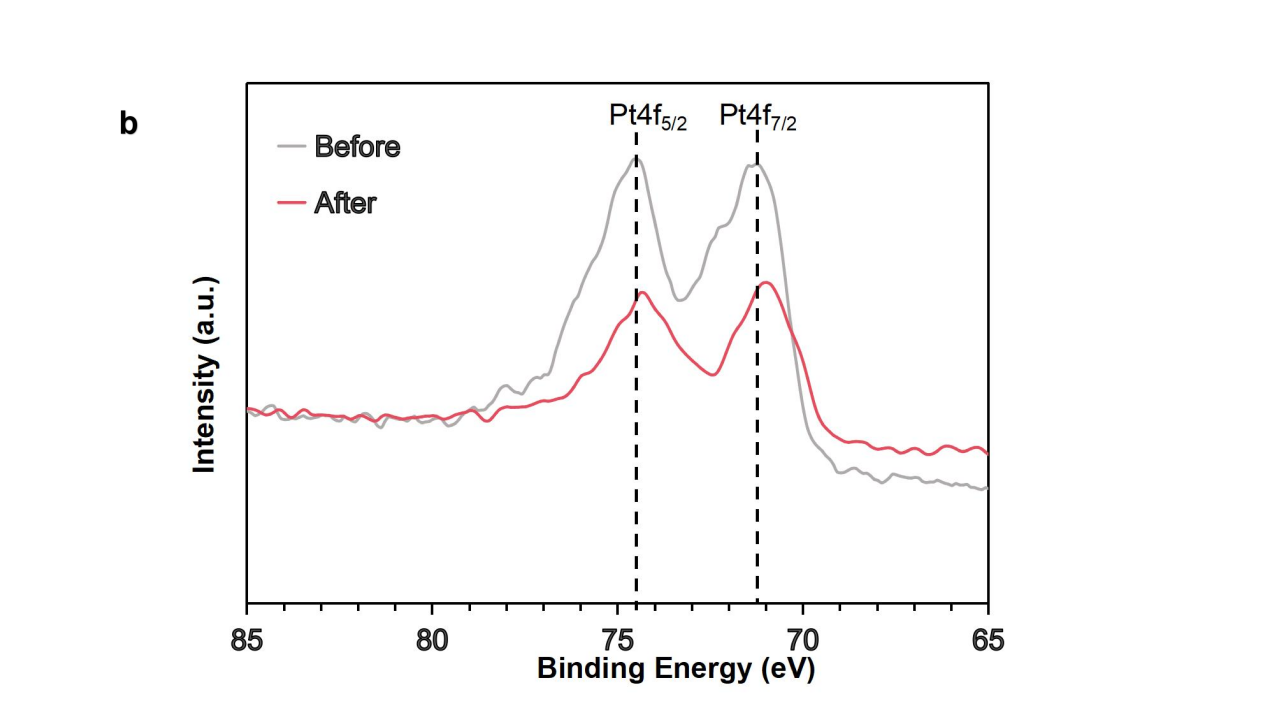


**Fig.S1** XPS scans before and after 1h photoelectrochemical process of the (a) Si 2p and (b) Pt 4f peak


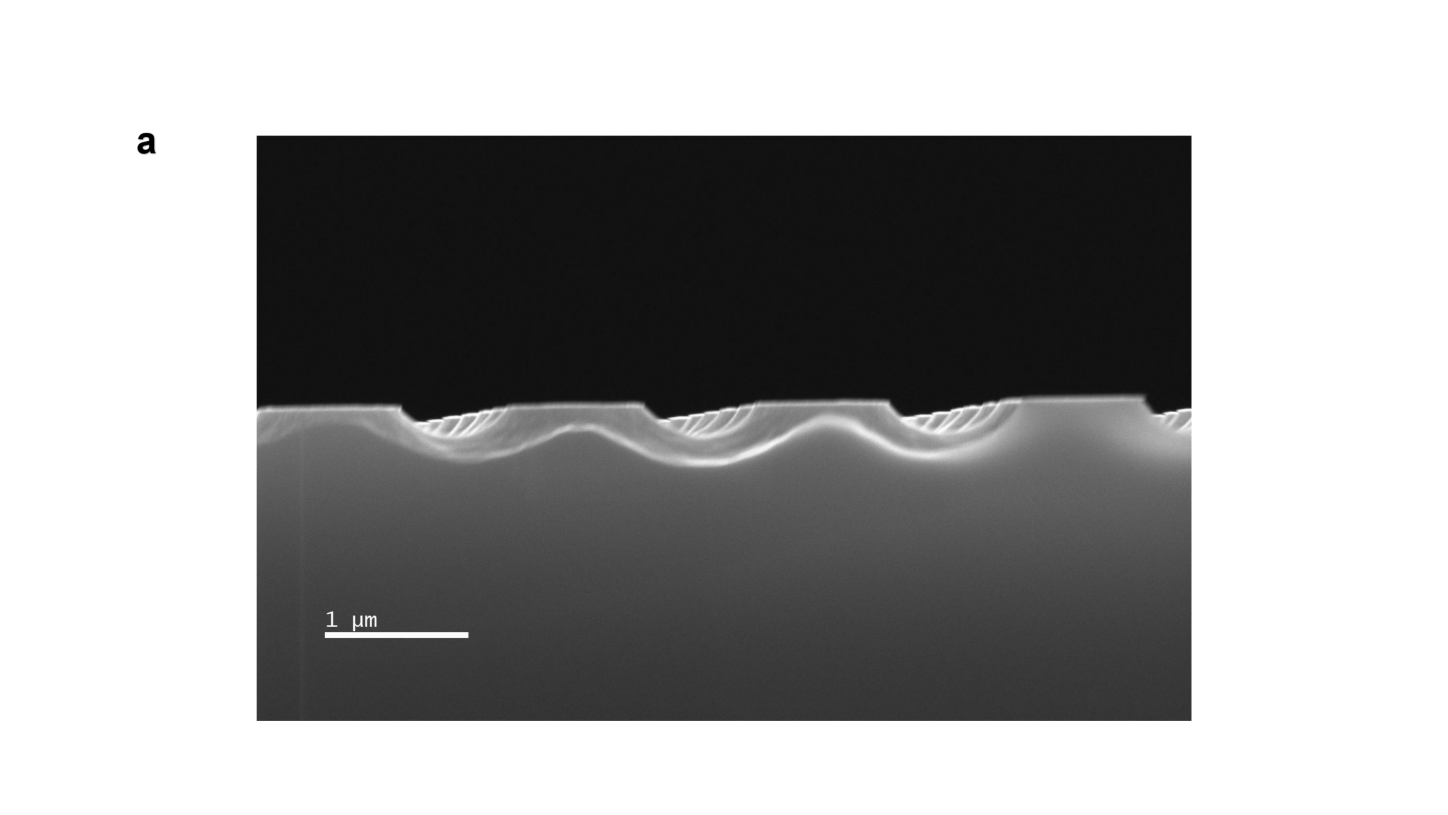

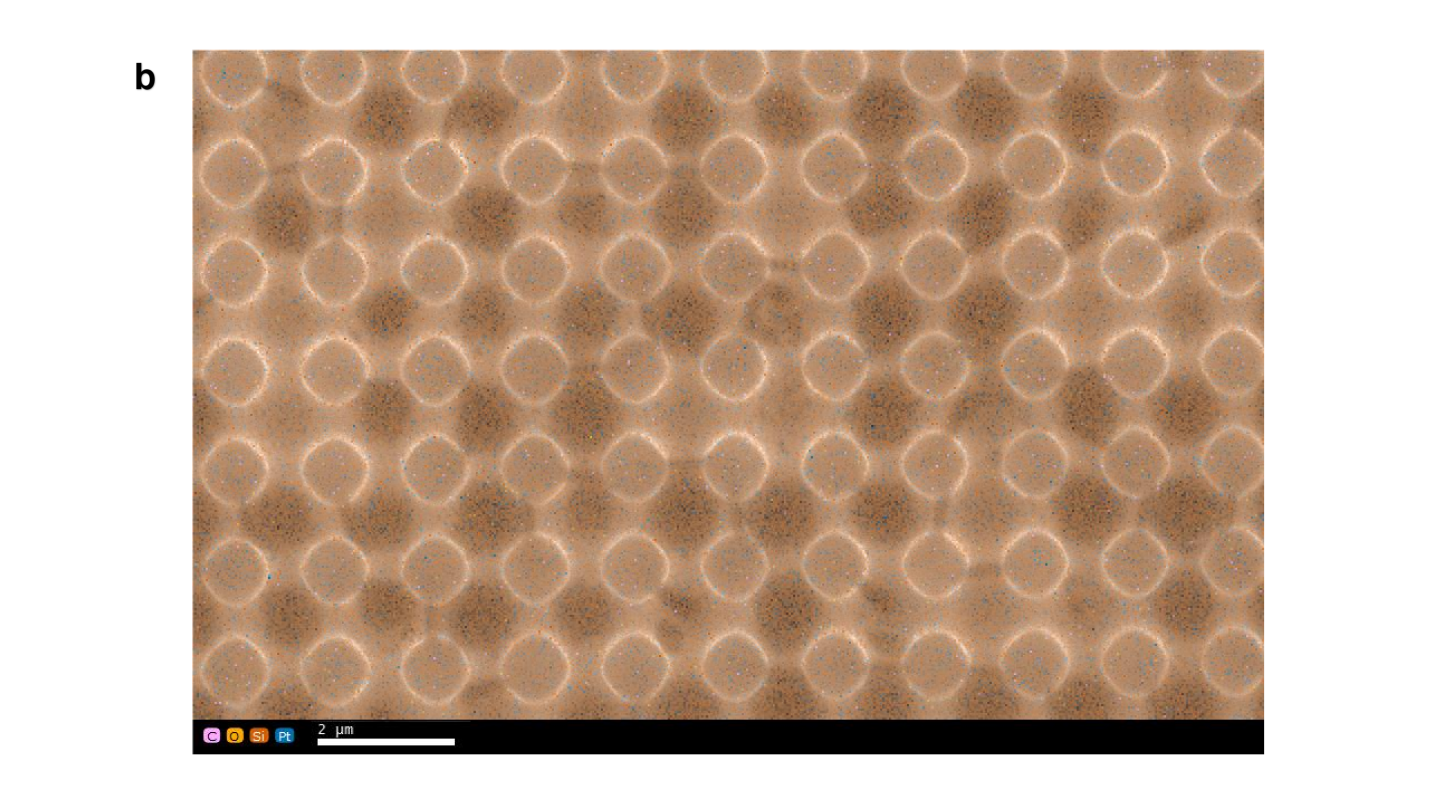


**Fig.S2** (a) Cross section SEM image of the SiPC. (b) EDS mapping of the SiPC after electrodeposition of Pt nanoparticles.


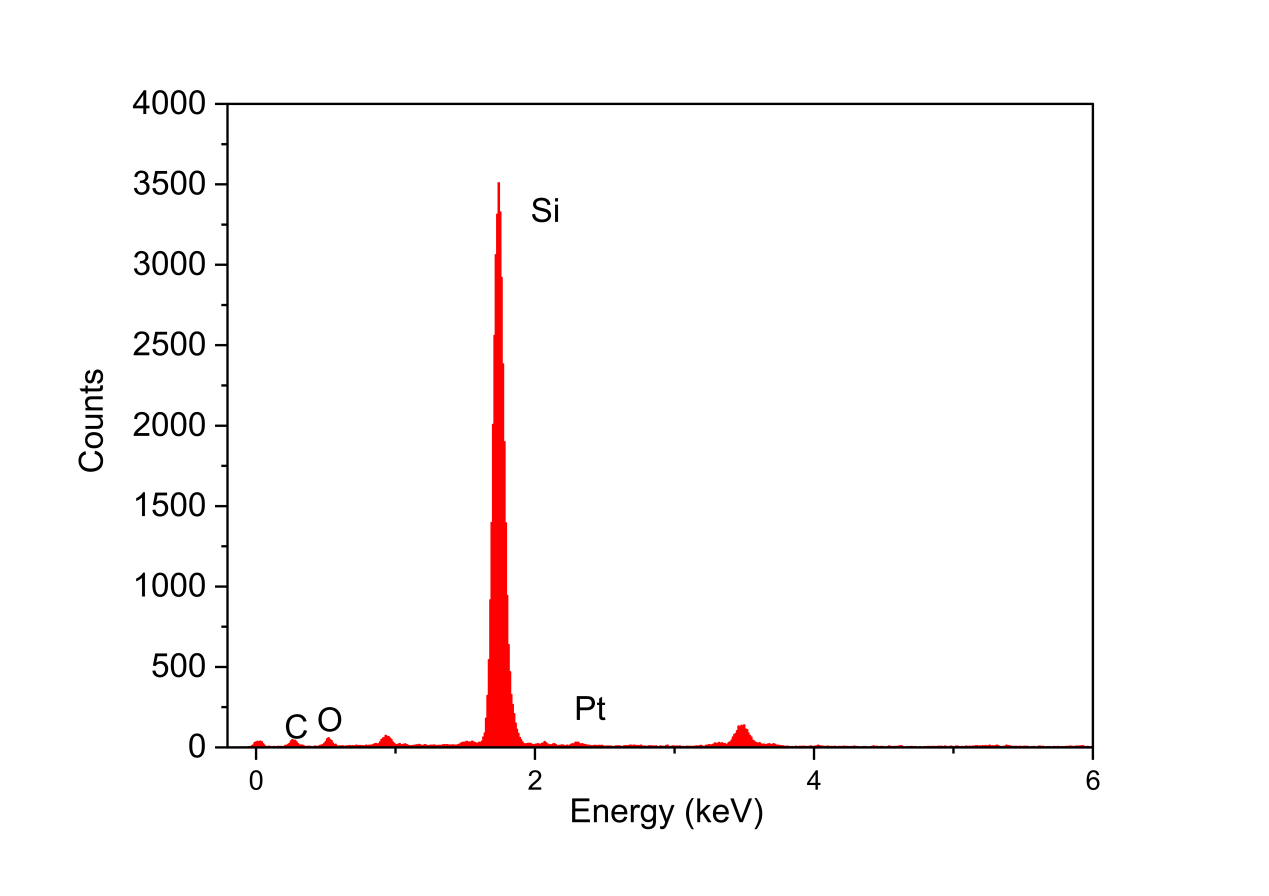


**Fig.S3** EDS analysis of Pt/SiPC sample scraped off from the Si substrate.


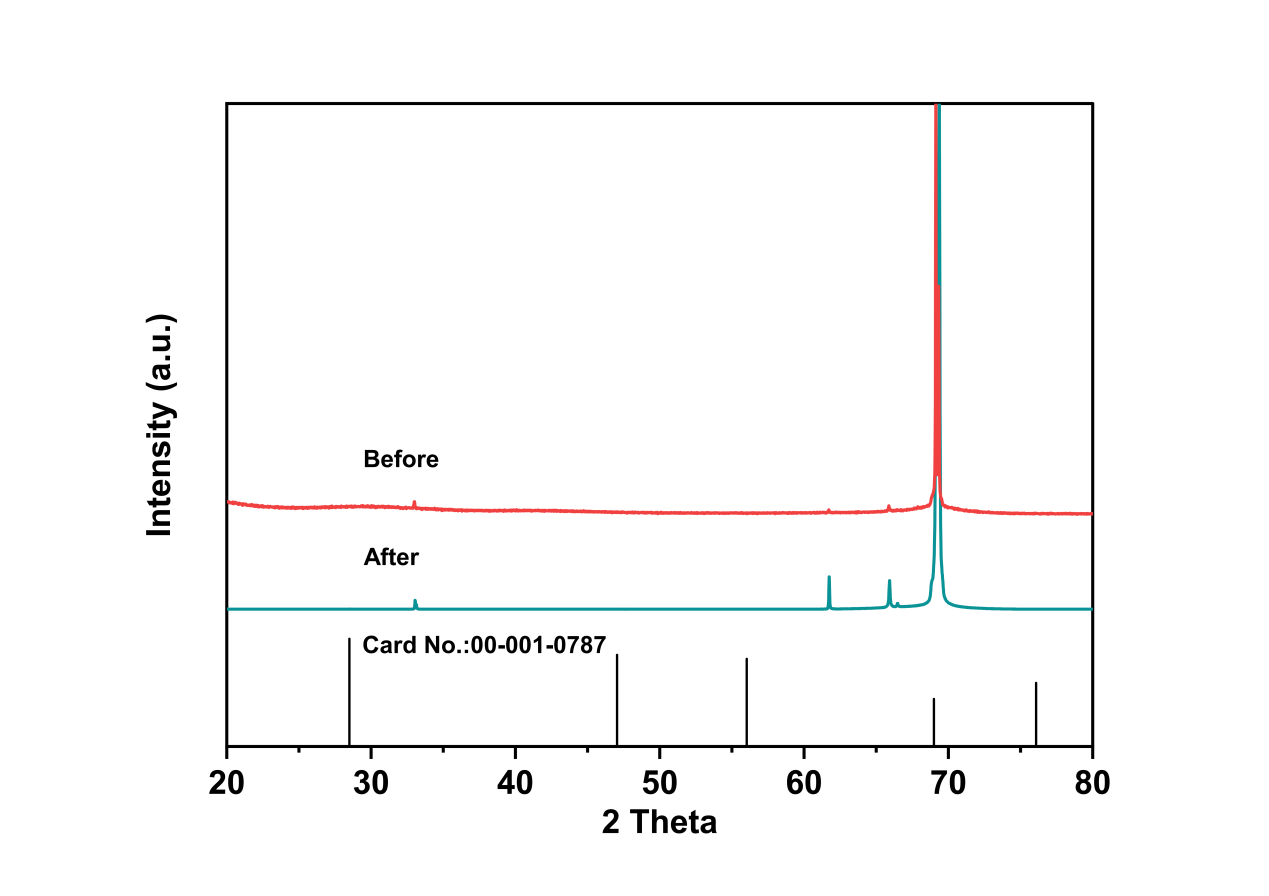


**Fig.S4** XRD pattern of the SiPC photocathode before and after PEC process.


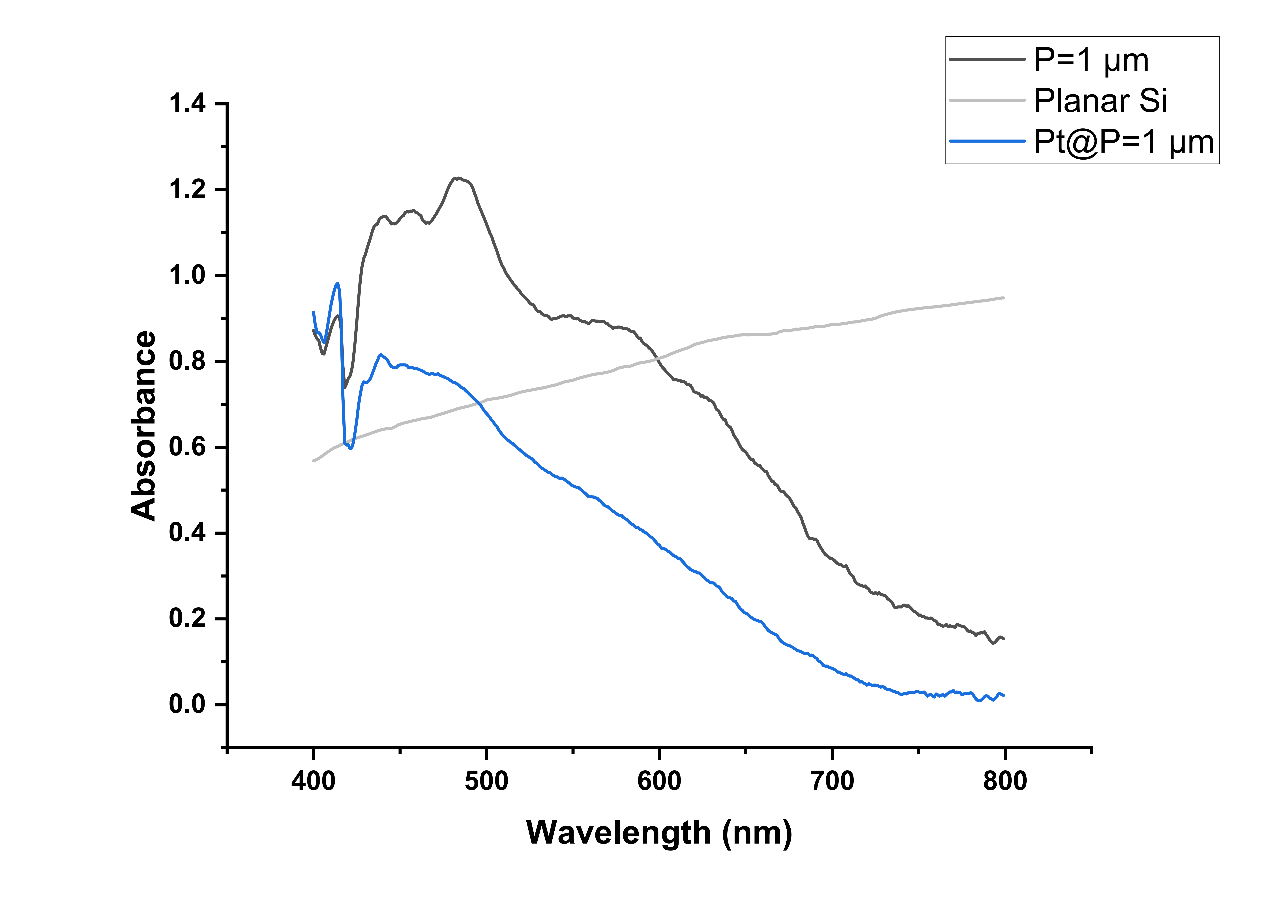


**Fig.S5** The absorption spectrum of Pt@SiPC. When compared to the sample without Pt deposited, there is a decrease in the absorbance. However, the period-dominated absorption peak remains significant, indicating the feasibility of using photonic crystals and metal nanoparticles in combination.
